# A radioligand for in vitro autoradiography of CSF1R in post-mortem CNS tissues

**DOI:** 10.1186/s13550-024-01133-2

**Published:** 2024-08-26

**Authors:** Catherine A. Foss, Ravi Naik, Deepankar Das, Hyojin Cha, Il Minn, Andrew Hall, Paige Finley, Sophia Jiang Wu, Yong Du, Robert F. Dannals, Martin G. Pomper, Andrew G. Horti

**Affiliations:** 1 The Russell H. Morgan Department of Radiology and Radiological Science, Johns Hopkins University, 1550 Orleans St. CRB2 493, Baltimore, MD 21228 USA; 2 Department of Pediatrics, Center for Infection and Inflammation Imaging Research, Johns Hopkins University, Baltimore, MD USA

**Keywords:** Colony stimulating factor 1 receptor, CD115, Alzheimer’s dementia, Inflammation imaging, B_max_

## Abstract

**Background:**

Reactive microglia and recruited peripheral macrophages contribute to the pathogenesis of Alzheimer’s dementia (AD). Monocytes, macrophages and microglia all express the marker colony-stimulating factor 1 receptor (CSF1R). 4-Cyano-N-(4-(4-methylpiperazin-1-yl)-2-(4-methylpiperidin-1-yl)phenyl)-1H-pyrrole-2-carboxamide (**1**) is a high-affinity antagonist for CSF1R. We report the radiosynthesis of both [^3^H]**1** and [^11^C]**1**. The PET imaging properties of [^11^C]**1** in mice and baboon were investigated. [^3^H]**1** was studied in B_max_ measurement in post-mortem autoradiography in the frontal cortex, inferior parietal cortex and hippocampus from donors diagnosed with AD and age-matched controls. In vitro binding affinity of **1** was measured commercially. Nor-methyl-**1** precursor was radiolabeled with [^11^C]iodomethane or [^3^H]iodomethane to produce [^11^C]**1** and [^3^H]**1**, respectively. Ex vivo brain biodistribution of [^11^C]**1** was compared in normal mice versus lipopolysaccharide-administered (LPS) murine model of neuroinflammation. Dynamic PET imaging was performed in a healthy male *Papio anubis* baboon. Post-mortem autoradiography with [^3^H]**1** was performed in frozen sections using a standard saturation binding technique.

**Results:**

Compound **1** exhibits a high in vitro CSF1R binding affinity (0.59 nM). [^11^C]**1** was synthesized with high yield. [^3^H]**1** was synthesized similarly (commercially). Biodistribution of [^11^C]**1** in healthy mice demonstrated moderate brain uptake. In LPS-treated mice the brain uptake of [^11^C]**1** was ~ 50% specific for CSF1R. PET/CT [^11^C]**1** study in baboon revealed low brain uptake (0.36 SUV) of [^11^C]**1**. Autoradiography with [^3^H]**1** gave significantly elevated B_max_ values in AD frontal cortex versus control (47.78 ± 26.80 fmol/mg vs. 12.80 ± 5.30 fmol/mg, respectively, *P* = 0.023) and elevated, but not significantly different binding in AD hippocampus grey matter and inferior parietal cortex (IPC) white matter.

**Conclusions:**

Compound **1** exhibits a high in vitro CSF1R binding affinity. [^11^C]**1** specifically labels CSF1R in the mouse neuroinflammation, but lacks the ability to efficiently cross the blood–brain barrier in baboon PET. [^3^H]**1** specifically labels CSF1R in post-mortem human brain. The binding of [^3^H]**1** is significantly higher in the post-mortem frontal cortex of AD versus control subjects.

**Supplementary Information:**

The online version contains supplementary material available at 10.1186/s13550-024-01133-2.

## Introduction

Damaged neurons, neurofibrillary tangles and amyloid-beta deposits provide stimuli for inflammation in the brains of patients with Alzheimer’s disease (AD) [1, 2]. The activated microglia in neuroinflammation of AD is associated with upregulation of complement, cytokines, acute phase reactants, and other inflammatory mediators [3, 4]. Translocator Protein (TSPO) has been a popular target for neuroimaging, including in AD. TSPO is a diagnostic target only and binding of radioligands is often dependent upon a patient’s genotype [5]. Growth and survival of microglia are dependent on signaling through the colony stimulating factor-1 receptor (CSF1R/CD115), a biomarker for both detection and therapy [6]. Previous research demonstrated an imbalance in CSF1R signaling in AD [7]. Several reports have established upregulation of CSF1R and its cognate ligand CSF1 in post-mortem brain in AD [7–9]. Post-mortem studies showed an increase in CSF1R (120–190%) and CSF1 (70–500%) expression in the temporal cortex of AD subjects versus age-matched controls [7, 10–12]. Autoradiography studies of CSF1R in healthy and AD brain would be important for studying the role of CSF1R in the disease. A PET radioligand for imaging of CSF1R, [^11^C]CPPC, demonstrated a degree of specific binding in postmortem human brain tissue of individuals with AD [13]. Further research demonstrated that [^3^H]CPPC may have utility as a radioligand tool for the evaluation of peripheral targets and screening of CSF1R inhibitors, but it has limited utility as an in vivo CNS imaging probe [14]. The goal of this study was to develop a radiotracer for specific imaging of CSF1R in post-mortem brain tissue.

The present study describes the synthesis and radiosyntheses of [^3^H]**1** and [^11^C]**1**, a more polar analog of CPPC [13–17] with higher binding affinity and radiological stability for in vitro studies. [^3^H]**1** is suitable for quantification of CSF1R concentration (B_max_) in post-mortem CNS autoradiography. We also demonstrated that [^11^C]**1** specifically labeled CSF1R in the LPS-treated mouse brain, but the total brain uptake was moderate. Brain uptake of [^11^C]**1** in healthy baboon, however, is low.

## Results

### Chemistry

**Scheme 1:**
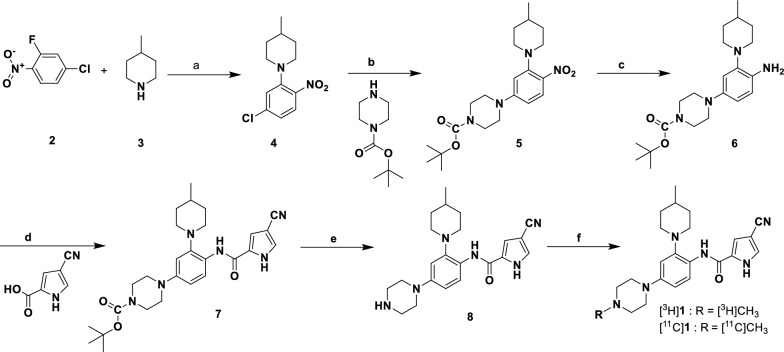
Synthesis of [^3^H]**1** and [^11^C]**1**

Precursor **8** for radiolabeling was synthesized as shown on the Scheme 1. Radiosynthesis of [^11^C]**1** was carried out in one step with radiochemical yield of 35%, specific radioactivity of = 426–633 GBq/µmol (11.5–17.1 Ci/µmol) by treating the precursor **8** with [^11^C]CH_3_I (Scheme 1). [^3^H]**1** was synthesized under the similar reaction conditions with [^3^H]CH_3_I (Scheme 1).

Stability of [^3^H]**1** for autoradiography experiments: The rate of decomposition of the [^3^H]**1** stock solution in alcohol was ~ 4% for the first 4 months when stored at -20 °C. After the stock solution of [^3^H]**1** was diluted with water in a ratio of 1:1000, it was stable for 8 h at room temperature.

**Reagents and conditions:** (a) Ethanol, 0 °C to rt, 0.5 h, 96%; (b) 110 °C, 12 h, K_2_CO_3_, DMSO, ~ 90%; (c) Zn, NH_4_Cl, THF/MeOH/H_2_O, reflux, 1 h, 90%; (d) 4-cyano-1H-pyrrole-2-carboxylic acid, HATU, DIPEA, DMF, 12 h, 88%; (e) TFA, MC, rt, 12 h, 90%; f) [^3^H]CH_3_I or [^11^C]CH_3_I DMF, 80 °C.

### In vitro binding assay (KINOME KdELECT scan, Eurofins)

Dissociation constants (*K*_d_) under the same in vitro assay conditions were determined commercially for **1** and CPPC (Table 1).

**Table 1:** CSF1R human in vitro binding assay of 1 and CPPC

Compound	Kd, nM
	0.59 ± 0.11 (n = 4)
	8.48 ± 5.02 (n = 8)

### Mouse studies with [^11^C]1

The regional brain uptake of [^11^C]**1** in control CD1 male mice at three time points after injection of radiotracer is shown in Table 2. A peak uptake value of 2.15% percent injected dose per gram (ID/g) tissue was seen in the frontal cortex in 2 min after the radiotracer injection with decline thereafter. The uptake in other regions studied here was lower.

**Table 2:** Regional distribution of [^11^C]**1** in CD1 ex vivo mouse brain (mean %ID/g tissue ± SD, n = 3)

	2 min	30 min	60 min
Frontal cortex	2.15 ± 0.44	1.49 ± 0.17	1.13 ± 0.08
Hippocampus	1.63 ± 0.31	1.32 ± 0.10	1.01 ± 0.17
Cerebellum	2.08 ± 0.39	1.39 ± 0.04	0.95 ± 0.09
Brain stem	1.78 ± 0.48	1.32 ± 0.10	0.92 ± 0.11
Rest of brain	1.81 ± 0.34	1.31 ± 0.10	0.90 ± 0.06

### Ex vivo biodistribution of [^11^C]1 in LPS-induced murine models of neuroinflammation

**Fig. 1:**
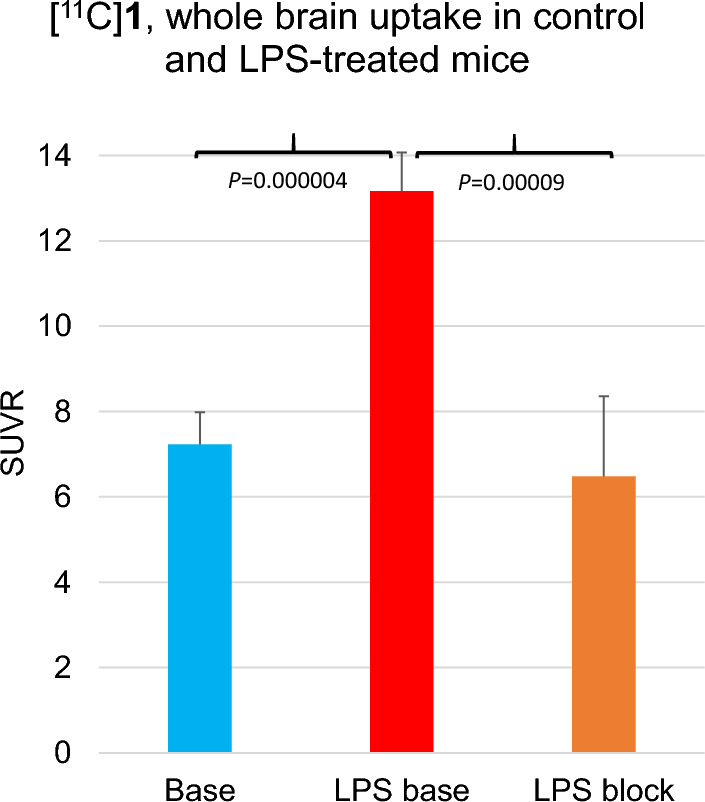
Brain uptake of CSF1R radiotracer [^11^C]1 in control mice (Base); LPS-treated mice (LPS base); and LPS-treated mice, blocking with CSF1R inhibitor (LPS block). The time point was 45 min after the radiotracer injection. The LPS treatment was IP, 10 mg/kg. Data are mean SUVR ± SD (n = 6). Blocker (CPPC, 1 mg/kg, IP) was injected 5 min before [^11^C]1 (0.1 mCi, IV). Statistical analysis: comparison of LPS-baseline versus control or LPS-block, ANOVA

Studies were performed in a murine LPS-induced neuroinflammation model [18, 19] under the conditions described previously [20]. The percentage of the standardized uptake value (%SUV) uptake in the whole brain at 40 min after injection was corrected for blood radioactivity as SUV^whole brain^/SUV^blood^ = SUV ratio (SUVR) (Fig. 1). The SUVR was significantly greater in LPS-treated mice than in controls. Blocking with CPPC (1 mg/kg) significantly decreased the uptake in the LPS-treated mice to the control level (LPS base/LPS block ratio = 2.03).

### Baboon PET with [^11^C]1

**Fig. 2:**
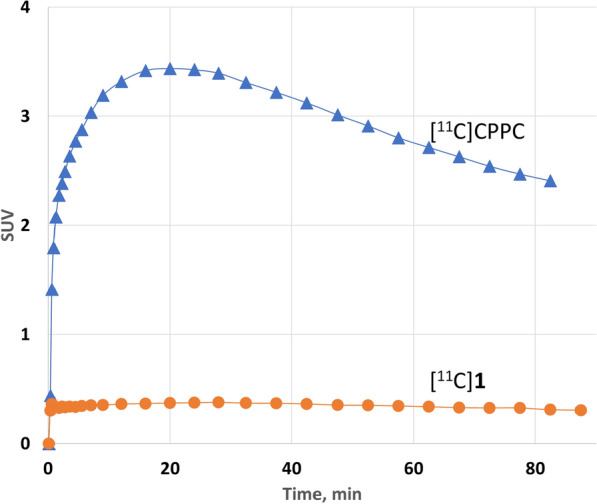
[^11^C]JHU11761 and [^11^C]CPPC whole brain time-uptake curves in control baboon

Dynamic PET/MRI [^11^C]**1** scan in a control baboon demonstrated low brain uptake with a peak of 0.36 SUV at 30 min after injection that was much lower than the previous studies showed for [^11^C]CPPC [13] (Fig. 2, Suppl Fig. 3).

Radiometabolite analysis of baboon blood showed that [^11^C]**1** was metabolized to two hydrophilic radiometabolites. The metabolism rate of [^11^C]**1** in this study was lower (16%) than was previously observed for [^11^C]CPPC [13] (75%) at 90 min post injection.

**B**_**max**_** measurements in post-mortem specimens of AD and non-AD control brain.** B_max_ measurements were completed in frozen sections of inferior parietal cortex (IPC), frontal cortex (FCTX) and hippocampus (HIPP) and are displayed in Tables 3, 4 and 5, respectively.

**Table 3:** Bmax measurements in IPC in control and AD

BRC#/ (IPC)	FDx	CERAD	BRAAK	sex	age	race	PMD	GM	WM
1517	C	A	2	F	71	W	16	14.24	1.53
1873	C	0	0	M	47	W	22	19.36	10.11
1615	C	0	0	M	72	W	20	9.60	3.39
2755	C*	A	3	F	100	W	8	18.69	3.21
1898	AD	C	5	M	79	W	10.5	14.05	4.12
2242	AD	C	5	M	62	W	19	10.07	3.43
1921	AD	C	6	F	72	W	10	22.41	6.61
1912	AD	C	6	F	62	W	11	9.82	2.15
2059	AD	C	6	M	76	W	15	7.41	0.91
2463	AD	B	4	F	84	W	16	39.71	30.46

Bmax fmol/mg sites of [^3^H]1 (CSF1R) in inferior parietal cortex

^*^Intermediate AD; FDx, functional diagnosis; CERAD, Consortium to Establish a Registry for Alzheimer’s Disease; PMD, post-mortem delay

Average GM AD: 17.45 ± 11.16 fmol/mg. Average WM AD: 7.27 ± 10.38 fmol/mg

Average GM C: 14.40 ± 4.88 fmol/mg. Average WM C: 5.01 ± 4.51 fmol/mg

**Table 4:** Bmax fmol/mg sites of [^3^H]1 (CSF1R) in frontal cortex

BRC# (FCTX)	FDx	CERAD	BRAAK	sex	age	race	PMD	GM	WM
2234	C	0	2	F	68	W	12	13.69	1.28
2228	C	0	1	M	64	W	28	14.05	4.12
2117	C	0	2	F	87	W	7	9.60	3.39
2775	C	0	2	F	88	W	9	22.27	6.57
2371	MLC*	0	3	M	77	A	6	9.80	2.15
2193	C	0	2	M	89	W	8.5	7.39	0.91
2534	AD	C	6	M	67	W	12	18.62	3.22
2641	AD	C	6	F	72	W	8	10.07	3.43
2663	AD	C	5	M	74	W	9	65.70	19.75
2669	AD	B	6	F	91	W	14	58.00	6.50
2709	AD	C	6	M	66	B	20	58.77	27.12

*MLC: metastatic lung cancer

Average GM AD: 47.78 ± 26.80 fmol/mg. Average WM AD: 15.40 ± 12.78 fmol/mg

Average GM C: 12.80 ± 5.30 fmol/mg. Average WM C: 3.07 ± 2.11 fmol/mg

**Table 5:** Bmax fmol/mg sites of [^3^H]1 (CSF1R) in hippocampus

BRC# (HIPP)	FDx	CERAD	BRAAK	sex	age	race	PMD	GM	WM
2775	C	0	2	F	88	W	9	59.32	17.03
2020	C	0	3	M	82	W	14.5	46.12	30.57
2021	C	0	4	M	99	B	24	52.71	30.90
2463	AD	B	4	F	84	W	16	45.69	29.18
1825	AD	C	6	F	79	W	5.5	61.10	12.09
2059	AD	C	6	M	76	W	15	72.14	34.19
1912	AD	C	6	F	62	W	11	88.55	33.99
1921	AD	C	6	F	72	W	10	70.10	32.13
2242	AD	C	5	M	62	W	19	79.33	33.06

Average GM AD: 69.49 ± 14.86 fmol/mg. Average WM AD: 29.22 ± 8.54 fmol/mg

Average GM C: 52.72 ± 6.60 fmol/mg. Average WM C: 26.17 ± 7.91 fmol/mg

**IPC**. Average gray matter (GM) uptake in AD IPC sections was 17.45 ± 11.16 fmol/mg and 7.27 ± 10.38 fmol/mg in white matter (WM), compared with 14.40 ± 4.88 fmol/mg (GM) and 5.01 ± 4.51 fmol/mg (WM) in control.

**FCTX**: In FCTX sections, AD GM averaged 47.78 ± 26.80 fmol/mg, while AD WM averaged 15.40 ± 12.78 fmol/mg. Control FCTX GM averaged 12.80 ± 5.30 fmol/mg, while control WM averaged 3.07 ± 2.11 fmol/mg.

**HIPP**. AD HIPP GM values averaged 69.49 ± 14.86 fmol/mg, while WM averaged 29.22 ± 8.54 fmol/mg. Control HIPP GM averaged 52.72 ± 6.60 fmol/mg, while WM averaged 26.17 ± 7.91 fmol/mg.

Figure 4 shows box-and-whisker plots of aggregate B_max_ values for AD cases and control for each brain region. Only B_max_ values for AD versus control FCTX reached significance (*P* < 0.05) while AD versus control HIPP GM values were just outside of significance (*P* = 0.052).

**Fig. 3:**
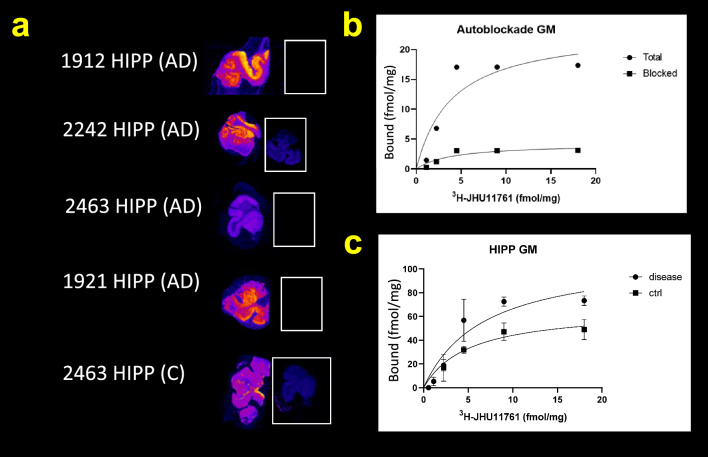
Autoradiograms of selected cases in hippocampus with Bmax tracer-only, autoblockade and saturation binding curves for total, blocked and specific binding. Saturation binding in selected cases is shown as maximum radioligand concentration binding (**a**). 10 µM unlabeled autoblockade with 657 pM radioligand binding is shown in the white boxes (a, right). Blockade was nearly quantitative for most cases and very high for others. A single control case showing total and blockade binding (**b**). Average saturation binding values in GM for HIPP cases (**c**)

Figure 3 shows individual autoradiographs of hippocampus binding (A), blockade (white boxes), an individual case showing total and blockade binding (B) and averaged saturation binding for GM in hippocampus (C).

## Discussion

Targets of interest for CNS neuroinflammation that are under active investigation include TSPO, P2X purineurgic receptor 7 (P2X7), monoamine oxidase-B (MAO-B), cyclooxygenase-1 (COX-1) and cyclooxygenase-2 (COX-2)[21]. Each of those targets is flawed due to either off-target expression (all of the above) or radiotracer difficulties such as many early radiometabolites or poor reference tissue availability [21]. TSPO is by far the most studied biomarker for CNS inflammation using an ever-evolving arsenal of radioligands designed to defeat genotype requirements and off-target binding. The biggest limitation, however, is that in humans, TSPO appears to be a surrogate biomarker whose connection with inflammation has been called into question [22]. Also in microglia, CSF1R expression has become a biomarker of great interest for CNS and peripheral inflammation as both a direct imaging and a therapeutic target. We previously reported the radiosynthesis and validation of [^11^C]CPPC, a small molecule radioligand with high affinity for CSF1R [20]. We identified in the literature [23] an analog of CPPC, namely 4-cyano-N-(4-(4-methylpiperazin-1-yl)-2-(4-methylpiperidin-1-yl)phenyl)-1H-pyrrole-2-carboxamide (**1**). Both unlabeled CPPC and **1** were originally synthesized by Illig et al. (see compounds 24 and 8, respectively [23]) with equal CSF1R functional potency (IC_50_ = 0.8 nM [23]). Functionally, compound **1** also inhibits several other kinases including (Kit, Axl, TrkA, FLT3 and IRKβ, but with five times lower potency (IC_50_ = 3.5–83 nM) than that for CSF1R [23]. Our in vitro recombinant kinase inhibition binding assay demonstrated that **1** exhibited at least an order of magnitude higher binding affinity (0.59 nM) than that of CPPC (Table 1). We report here efficient radiosyntheses of both [^11^C]**1** and [^3^H]**1**(Scheme 1) using conventional radiomethylation of corresponding nor-methyl precursor **8** that was synthesized in this study..

Ex vivo studies with [^11^C]**1**. [^11^C]**1** radioligand displays a 50% specific CSF1R binding in a murine model of neuroinflammation, but the total mouse brain uptake was moderate (~ 2% ID/g tissue). Baseline PET/[^11^C]**1** imaging in baboon brain demonstrated much lower radioactivity uptake (0.36 SUV) compared with [^11^C]CPPC (3.5 SUV), making [^11^C]**1** an unlikely candidate for CNS PET studies even though [^11^C]**1** was much more metabolically stable than [^11^C]CPPC.

**Fig. 4:**
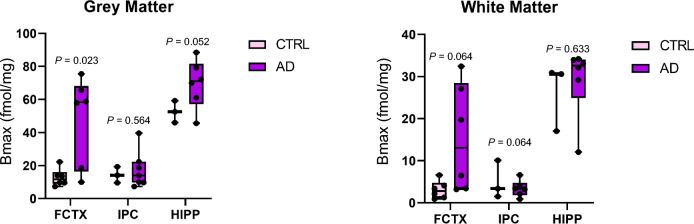
Box and whisker plots of Bmax values for all tissues. Values, ranges and significance for Bmax measurements in Grey matter and white matter across FCTX, HIPP and IPC. Grey matter uptake is higher in FCTX and HIPP among AD donors while WM uptake appears to be less specific

Post-mortem in vitro studies with [^3^H]**1**. Because the radiochemical stability test demonstrated that [^3^H]**1** stock solution in alcohol is stable for months, this tritiated stock is suitable for longstanding biological binding assays, such as is needed for tissue Bmax assays of numerous donors and exposures. In order to quantify CSF1R densities in human brain sections, in vitro Bmax measurements were conducted with [^3^H]**1** in post-mortem human brain sections from individuals with either an AD diagnosis or age-matched (non-AD) controls in inferior parietal cortex, frontal cortex and hippocampus. Autoblockade was carried out to determine specific from non-specific binding. The values obtained (Tables 3, 4, 5, Fig. 4) support elevated CSF1R densities in brain regions from patients with AD in grey matter and less so in white matter, likely due to some non-specific myelin adsorption.

Frontal cortex Tau tangles, Aβ plaque accumulation and inflammation are reported to be involved in both mild cognitive impairment (MCI) and AD and begin early (Braak I, II)[24]. PET studies in patients with Frontal Temporal Dementia using TSPO ligand ^11^C-(R)-PK11195 show increased radiotracer uptake in FCTX and temporal regions according to clinical phenotype and are supported by immunohistochemistry (IHC) relative to healthy controls [24]. However, studies reported using [^3^H]PK11195 and [^3^H]PBR28 in vitro autoradiography found no significant difference in binding in FCTX among post-mortem AD and control brain sections [25], supporting a lack of TSPO specificity in human tissues. [^3^H]**1** in vitro uptake in FCTX reported here reached significance in GM (*P* = 0.023) but not quite in WM (0.064) (Fig. 4). Averages are noisy and would have benefitted from more cases.

Decreases in hippocampal volume and activation of microglia have long been recognized in AD. Tissue loss begins early in disease. Similarly, the hippocampus is also known as a brain region that is inflamed early on in AD. IHC staining of post-mortem subiculum from AD against TSPO and Iba1 confirmed higher TSPO expression in subiculum from demented donors that line up with microglial staining (Iba1) versus non-demented tissue sections [26]. In vivo clinical [^18^F]FEPPA PET has shown a positive correlation between hippocampal uptake and amnestic score while [^11^C]PBR28 PET uptake correlated with Aβ and phosphorylated Tau tangles (pTau) burden. Other published studies show correlations of various TSPO tracers with non-TSPO clinical features in AD in the hippocampus [27]. Reported here for CSF1R densities, box and whisker plots in Fig. 4 show significant difference in the frontal cortex between AD and control subjects. The differences in average GM uptake was elevated but insignificant due to the presence of outlier values in AD samples. It’s possible that age and potential comorbidities within some of the controls contributed to the small difference in Bmax values or this may be due to variable AD expression patterns among donors [28] or the use of anti-inflammatory medication prior to death. Similar Bmax values in AD and control hippocampus may reflect tissue loss. Inferior parietal cortex is considered to be one of the specific neuroimaging tissues in predicting the conversion of MCI to AD [29]. IPC in our studies did not show a difference between control and AD in CSF1R binding. AD begins in the medial temporal lobe before it spreads to the parietal lobes in mild AD and beyond [22]. In vivo studies by others in AD versus control using [^11^C]-(*R*)-PK11195 PET to detect TSPO densities in microglia and astrocytes, showed a higher uptake in AD IPC versus healthy control [23] while several other TSPO-targeted studies with the same and other TSPO-specific radiotracers were unable to discern differences between AD and healthy controls in this subregion [24]. Once mild cognitive impairment (MCI) has been reached, tissue loss in the inferior parietal lobe begins and continues in AD [25]. This loss of tissue may paradoxically contribute to reduced Bmax values, possibly as inversely proportional to disease state. A study with [^11^C]FEPPA TSPO PET found higher radiotracer uptake in milder disease [26].

Figure 3 shows a high degree of specific binding in GM (Fig. 3B), but predictably less-so in white matter (Supplementary Fig. 2). Our binding data fit saturation binding curves (Fig. 3, Supplemental Figs. 1, 2) although there was a high degree of deviation in the averaged data throughout the sets. Post-mortem donors are different from each other, have co-morbidities and are likely to have been medicated with any number of pharmacotherapies prior to death. Medication data were not available for this cohort and it is important to recognize that CSF1R upregulation with subsequent microglial activation takes place in all chronic neurodegenerative disorders [30]. This manuscript therefore delivers trends in CSF1R radioligand binding in those dying with AD and in age-matched controls.

Peripheral inflammation plays a central role in initiating and perpetuating AD [31], including chronic GI inflammation. Neuroinflammation from chronic and acute sources are often harmful [32]. We have shown here B_max_ values from a collection of post-mortem tissues probed with [^3^H]**1** in which gray matter radioligand uptake from FCTX is significantly higher than in control and gray matter uptake in HIPP was nearly significantly higher than in control with limited cases. White matter values in FCTX and IPC came close to significance in comparison to controls. A sample size has limited conclusions but individual case Bmax values demonstrate that inflammation occurs both within and outside of an AD diagnosis and rather than focusing on a diagnostic label, instead we may enter the molecular age by identifying and treating inflammation as both a potentiator and perpetuator of disease from any label.

## Conclusion

Compound **1** was confirmed to bind tightly to CSF1R in in vitro inhibition assay (0.59 ± 0.11 nM). [^11^C]**1** exhibited poor in vivo brain uptake, making it unsuitable for in CNS PET studies. [^3^H]**1** exhibited specificity and selectivity for CSF1R in autoradiography studies, making it a useful tool for post-mortem studies. The binding of [^3^H]**1** is significantly higher in the post-mortem frontal cortex of AD subjects versus control subjects.

## Methods

All reagents were used directly as obtained commercially unless otherwise noted. Reaction progress was monitored by thin-layer chromatography (TLC) using silica gel 60 F254 (0.040 − 0.063 mm) with detection by UV. All moisture-sensitive reactions were performed under an argon atmosphere using oven-dried glassware and anhydrous solvents. Column flash chromatography was carried out using BDH silica gel 60 Å (40 − 63 micron). Analytical TLC was performed on plastic sheets coated with silica gel 60 F254 (0.25 mm thickness, E. Merck, Darmstadt, Germany). ^1^H NMR spectra were recorded with a Bruker-500 NMR spectrometer at nominal resonance frequencies of 500 MHz in CDCl_3_, CD_3_OD or DMSO-*d*_*6*_ (referenced to internal Me_4_Si at *δ* 0 ppm). The chemical shifts (*δ*) were expressed in parts per million (ppm). High-resolution mass spectra were recorded utilizing electrospray ionization (ESI) at the University of Notre Dame Mass Spectrometry facility. A dose calibrator (Capintec 15R) was used for all radioactivity measurements. Radiolabeling with ^11^C was performed with a modified GE MicroLab radiochemistry module. HPLC purification and analysis of radiolabeled compounds were performed with Agilent 1260 Infinity System with UV detector and a Bioscan Flow-Count interface with a NaI radioactivity detector. The experimental animal protocols were approved by the Animal Care and Use Committee of the Johns Hopkins Medical Institutions. Standard compound, 4-cyano-N-(4-(4-methylpiperazin-1-yl)-2-(4-methylpiperidin-1-yl)phenyl)-1H-pyrrole-2-carboxamide, **1** was synthesized as described previously (see compound 8[23]). CPPC (5-cyano-N-(4-(4-methylpiperazin-1-yl)-2-(piperidin-1-yl)phenyl)furan-2-carboxamide) was synthesized as described previously (see compound 24[23]). In vitro CSF1R human RTK kinase binding assay was performed commercially (Eurofins DiscoverX Corporation).

**1-(5-Chloro-2-nitrophenyl)-4-methylpiperidine (4):** To a cooled (0 ^0^C) solution of 1.0 g (10.0 mmol) of 4-chloro-2-fluoronitrobenzene in 15 mL of EtOH was added 1.01 mL (30.0 mmol) of 4-methylpiperidine dropwise over 5 min. The solution stirred at 0 °C for 10 min and then at 23 ^0^C for 30 min. The mixture was poured into water (225 mL) and extracted with EtOAc (2 × 30 mL). The combined extracts were washed with saturated aq NaHCO_3_ and brine (30 mL each) and then dried over Na_2_SO_4_ and evaporated to get the crude compound. The resulting residue was purified by silica gel column chromatography (Hexane:EtOAc = 95:5) to give 1-(5-chloro-2-nitrophenyl)-4-methylpiperidine as a yellow solid (1.4 g, 96% yield). ^1^H NMR (500 MHz, CDCl_3_) *δ* 7.77 (d, *J* = 5.0 Hz, 1H), 7.13 (s, 1H), 6.93 (d, *J* = 10.0 Hz, 1H), 3.30–3.27 (m, 2H), 2.91–2.86 (m, 2H), 1.90–1.86 (m, 1H), 1.75–1.73 (m, 2H), 1.49–1.42 (m, 1H), 1.02 (d, *J* = 5.0 Hz, 3H).

***Tert-butyl 4-(3-(4-methylpiperidin-1-yl)-4-nitrophenyl)piperazine-1-carboxylate (5):*** To the mixture of 1-(5-Chloro-2-nitrophenyl)-4-methylpiperidine, **4** (1.0 g, 3.92 mmol) and *tert*-butyl piperazine-1-carboxylate (1.46 g, 7.85 mmol), in DMSO (10 mL) was added K_2_CO_3_ (1.62 g, 11.77 mmol). The reaction mixture was stirred at 110 °C for 12 h and then partitioned between EtOAc and brine. The organic layer was separated, dried over anhydrous MgSO_4_, filtered, and concentrated under a vacuum. The resulting residue was purified by silica gel column chromatography (Hexane:EtOAc = 3:7) to give *tert*-butyl 4-(3-(4-methylpiperidin-1-yl)-4-nitrophenyl)piperazine-1-carboxylate as a white solid (1.42 g, 89.8% yield). ^1^H NMR (500 MHz, CDCl_3_) *δ* 7.99 (d, *J* = 10.0 Hz, 1H), 6.38 (d, *J* = 10.0 Hz, 1H), 6.31 (s, 1H), 3.58 (t, *J* = 5.0 Hz, 4H), 3.34 (t, *J* = 5.0 Hz, 4H), 2.28 (t, *J* = 5.0 Hz, 2H), 2.78 (d, *J* = 10.0 Hz, 2H), 1.70 (d, *J* = 5.0 Hz, 2H), 1.55–1.51 (m, 3H), 1.47 (s, 9H), 1.00 (d, *J* = 5.0 Hz, 3H).

***Tert-butyl 4-(4-amino-3-(4-methylpiperidin-1-yl)phenyl)piperazine-1-carboxylate (6):*** To a mixture of tert-butyl 4-(3-(4-methylpiperidin-1-yl)-4-nitrophenyl)piperazine-1-carboxylate, **5** (1.2 g, 2.96 mmol), and NH_4_Cl (1.58 g, 29.6 mmol) in THF/MeOH/H_2_O (10:5:3) (20 mL), was added Zn dust (1.93 g, 29.6 mmol) at 90 ˚C, then the mixture was refluxed for 1 h. After completion of the reaction, the reaction mixture was filtered through Celite and partitioned between EtOAc and brine. The organic layer was separated, dried over anhydrous MgSO_4_, filtered, and concentrated *in vacuo*. The resulting residue was purified by silica gel column chromatography (CH_2_Cl_2_: MeOH = 9:1) to give *tert*-butyl 4-(4-amino-3-(4-methylpiperidin-1-yl)phenyl)piperazine-1-carboxylate as a brown solid (1.0 g, 90.0% yield).

***Tert-butyl 4-(4-(4-cyano-1H-pyrrole-2-carboxamido)-3-(4-methylpiperidin-1-yl)phenyl)piperazine-1-carboxylate (7):*** To the mixture of *tert*-butyl 4-(4-amino-3-(4-methylpiperidin-1-yl)phenyl)piperazine-1-carboxylate (0.5 g, 1.33 mmol), 4-cyano-1H-pyrrole-2-carboxylic acid (0.23 g, 1.60 mmol), HATU (0.61 g, 1.60 mmol), in DMF (10 mL) was added DIPEA (0.46 mL, 2.66 mmol). The reaction mixture was stirred at room temperature overnight and then partitioned between EtOAc and brine. The organic layer was separated, dried over anhydrous MgSO_4_, filtered, and concentrated under a vacuum. The resulting residue was purified by silica gel column chromatography (CH_2_Cl_2_: MeOH = 9:1) to give *Tert-*butyl 4-(4-(4-cyano-1H-pyrrole-2-carboxamido)-3-(4-methylpiperidin-1-yl)phenyl)piperazine-1-carboxylate as a yellow solid (0.58 g, 88.0% yield). ^1^H NMR (500 MHz, CDCl_3_) *δ* 10.40 (s, 1H), 8.99 (s, 1H), 8.26 (d, *J* = 5.0 Hz, 1H), 7.45 (s, 1H), 6.83 (d, *J* = 10.0 Hz, 2H), 6.73 (d, *J* = 5.0 Hz, 1H), 3.58 (t, *J* = 5.0 Hz, 4H), 3.10 (t, *J* = 5.0 Hz, 4H), 2.99 (t, *J* = 5.0 Hz, 2H), 2.72 (t, *J* = 10.0 Hz, 2H), 1.83 (d, *J* = 10.0 Hz, 2H), 1.55–1.51 (m, 3H), 1.49 (s, 9H), 1.07 (d, *J* = 5.0 Hz, 3H).

**4-Cyano-N-(2-(4-methylpiperidin-1-yl)-4-(piperazin-1-yl)phenyl)-1H-pyrrole-2-carboxamide (8):** To a solution of *tert-*butyl 4-(4-(4-cyano-1H-pyrrole-2-carboxamido)-3-(4-methylpiperidin-1-yl)phenyl)piperazine-1-carboxylate, **7** (0.5 g, 1.02 mmol) in methylene chloride (5 mL) was added trifluoroacetic acid (0.37 mL, 5.05 mmol) dropwise at 0 °C, and then, the mixture was stirred at room temperature for 12 h. After completion of the reaction, the reaction mixture was concentrated under reduced pressure. The resulting residue was purified by silica gel column chromatography (CH_2_Cl_2_: MeOH = 9:1) to give 4-cyano-N-(2-(4-methylpiperidin-1-yl)-4-(piperazin-1-yl)phenyl)-1H-pyrrole-2-carboxamide as a pale white solid (0.30 g, 78.4% yield). ^1^H NMR (500 MHz, MeOD) *δ* 10.45 (s, 1H), 8.98 (s, 1H), 8.24 (d, *J* = 5.0 Hz, 1H), 7.45 (d, *J* = 5.0 Hz, 1H), 6.83 (d, *J* = 10.0 Hz, 2H), 6.72 (d, *J* = 5.0 Hz, 1H), 3.15 (t, *J* = 5.0 Hz, 4H), 3.08 (t, *J* = 5.0 Hz, 4H), 2.99 (t, *J* = 5.0 Hz, 2H), 2.73 (t, *J* = 10.0 Hz, 2H), 1.84 (d, *J* = 10.0 Hz, 2H), 1.57 (s, 1H), 1.55–1.51 (m, 3H), 1.07 (d, *J* = 5.0 Hz, 3H).

### Radiosynthesis of 4-cyano-N-(4-(4-[^11^CH_3_]methylpiperazin-1-yl)-2-(4-methylpiperidin-1-yl)phenyl)-1H-pyrrole-2-carboxamide, [^11^C]1.

To a 1 mL V-vial, precursor, 4-cyano-N-(2-(4-methylpiperidin-1-yl)-4-(piperazin-1-yl)phenyl)-1H-pyrrole-2-carboxamide, **8** (1 mg) was added in solution of 0.2 mL of anhydrous DMF. [^11^C]Methyl iodide, carried by a stream of helium, was trapped in the precursor solution. The reaction was heated at 80 °C for 3.5 min, then quenched with 0.2 mL of water. The crude reaction product was purified by reverse-phase high performance liquid chromatography (HPLC) at a flow rate of 12 mL/min. The radiolabelled product [^11^C]**1** (t_R_ = 7–8 min) was fully separated from the precursor (t_R_ = 2.7 min) and collected in a solution of 0.3 g sodium ascorbate in 50 mL water. The aqueous solution was transferred through an activated Waters Oasis Sep-Pak Light cartridge (Milford, MA). After washing the cartridge with 10 mL saline, the product was eluted with 1 mL of ethanol through a 0.2 μM sterile filter into a sterile, pyrogen-free vial containing 4 mL saline, and 10 mL of 0.9% saline was added through the same filter. The final product, [^11^C]**1**, was analyzed by analytical HPLC to determine the radiochemical purity (> 99%) and specific radioactivity (15–23 Ci/µmol). The radiochemical purity of the final product did not change within 90 min after the end-of-synthesis.

**HPLC conditions for [**^**11**^**C]1**. Preparative HPLC: Column, XBridge C18, 10 × 250 mm (Waters, Milford, MA). Mobile phase: 39%:61% acetonitrile: triethylamine-phosphate buffer, pH 7.2. Flow rate: 12 mL/min, retention time 8 min. Analytical HPLC: Column, Luna C18, 10 micron, 4.6 × 250 mm (Phenomenex, Torrance, CA). Mobile phase: 55%:45% acetonitrile: 0.1 M aq. ammonium formate. Flow rate: 3 mL/min, retention time 3.7 min.

### Radiosynthesis of 4-cyano-N-(4-(4-[^3^H]methylpiperazin-1-yl)-2-(4-methylpiperidin-1-yl)phenyl)-1H-pyrrole-2-carboxamide, [^3^H]1

**[**^**3**^**H]1** was synthesized commercially (Novandi, Sweden) via the reaction of [^3^H]methyl iodide and precursor **8** (Scheme 1) that was prepared by our group. The specific activity of [^3^H]**1** was 83 Ci/mmol (3.07 TBq/mmol) and radiochemical purity was > 99%. [^3^H]**1** was formulated as a stock solution in ethanol (1 mCi/mL) that was stabilized with 0.01% sodium ascorbate.

**Stability of [**^**3**^**H]1** was determined by HPLC: retention time—6. 7 min. Column: Atlantis T3 C18 (100 × 4.6 mm, 3 µm), flow rate—0.8 mL/min. Solvent A—0.1% aqueous TFA; Solvent B—acetonitrile; gradient: 0 min—5% solvent B, 13 min—95% solvent B.

### In vitro assay

In vitro CSF1R human RTK kinase binding assay was performed commercially, assay # 87-0007-1104, KINOMEscan (KdELECT) (Eurofins DiscoverX Corporation). In brief, KINOME*scan*™ is based on a competition binding assay that quantitatively measures the ability of a compound to compete with an immobilized, active-site directed ligand.

## Mouse studies

Please see Supplemental Information for all murine in vivo methods

### Baboon PET scan

Dynamic PET scan was performed on a male baboon (*Papio anubis*, 27 kg) using the High Resolution Research Tomograph (CPS Innovations, Knoxville, TN) with an IV injection of 751 MBq (20.3 mCi) [^11^C]**1** [specific radioactivity: 518 TBq/mmol (14,000 Ci/mmol)]. PET imaging and data analysis and radiometabolite analysis of baboon arterial blood are described in detail in the SI Appendix.

### Baboon PET imaging methods

PET images were acquired using a CPS/CTI High Resolution Research Tomograph (HRRT), which has an axial resolution (FWHM) of 2.4 mm, and in plane resolution of 2.4–2.8 mm. The animal was anesthetized and handled as described previously [33]. The 90 min PET data were binned into 30 frames: four 15-s, four 30-s, three 1-min, two 2-min, five 4-min, and twelve 5-min frames. Images were reconstructed using the iterative ordered subset expectation maximization (OS-EM) algorithm (with six iterations and 16 subsets) with correction for radioactive decay, deadtime, attenuation, scatter and randoms [34]. The reconstructed image space consisted of cubic voxels, each 1.22 mm^3^ in size, and spanning dimensions of 31 cm × 31 cm (transaxially) and 25 cm (axially).

Blood samples were obtained via the arterial catheter at continually prolonged intervals throughout the 90 min scan (as rapidly as possible for the first 90 s, with samples acquired at increasingly longer intervals thereafter). Samples were centrifuged at 1200×*g* and the radioactivity in plasma were measured with a cross-calibrated gamma counter. Selected plasma samples (5, 10, 20, 30, 60, and 90 min) were analyzed with high performance liquid chromatography (HPLC) for radioactive metabolites in plasma as described above.

### Baboon PET data analysis

The image analysis and kinetic modeling were performed using software PMOD (v3.7, PMOD Technologies Ltd, Zurich, Switzerland). Dynamic PET images were first co-registered with the MRI images. A locally developed volume-of-interest (VOI) template, including 13 representative baboon brain structures, was then transferred to the animal’s MRI image. The VOIs included frontal and temporal gyrus, thalamus, hippocampus, caudate, putamen, amygdala, globus pallidus, insula, hypothalamus, cerebellum, corpus callosum, and white matter. Time activity curve (TAC) of each VOI was obtained by applying the VOI on PET frames.

Next, based on the TACs and the metabolite-corrected arterial plasma input functions, kinetic modeling was performed to quantitatively characterize the [^11^C]**1** binding in brain. For brain uptake, the primary outcome measure is the regional brain distribution volume (V_T_) of [^11^C]**1**, defined as concentration of the radiotracer in regional tissue relative to that in blood at equilibrium. Regional V_T_ is proportional to the receptor density in the defined VOI. Because we don’t anticipate any brain region to be devoid of specific [^11^C]**1** uptake, another commonly used outcome measure, namely, the non-displaceable binding potential (BPND), may not be obtained reliably.

For each VOI, V_T_ was calculated using both compartmental modeling and the Logan graphical method [35]. Results showed both one-tissue compartmental modeling and Logan method are suitable for analyzing the [^11^C]**1** PET data.

**B**_**max**_** measurements**. Unfixed, frozen human brain tissues were obtained from the JHU Brain Resource Center from deaths occurring from Alzheimer’s Dementia (AD) or non-AD controls. Tissues were cryosectioned to 16 µm thickness on charged glass slides and kept frozen at − 80 °C until use. Serially diluted solutions containing [^3^H]**1** in FBS (Sigma-Aldrich, St. Louis, MO) spanned 657 pM through 657 f*M* (corrected for serum protein binding). Each solution was applied to cognate slides and allowed to bind at ambient temperature for 10 min. Solutions were then rapidly aspirated off and the slides washed in ice cold PBS (St. Louis) for 2 min. The PBS was aspirated off and any remaining moisture removed. The dry slides were then loaded into a Hypercassette (Amersham RPN-11647) and exposed to a charged phosphorscreen (Cytiva, BAS-TR 2040) for about 5 days. The screen was scanned using a Typhoon 9500IP Phosphorimager (Molecular Dynamics) and data visualized and quantitated using ImageJ software (Sourceforge.net). ROIs were drawn over grey and then white matter to quantitate radioligand uptake and then transformed with a tritium scales standard (ARC, St. Louis, MO). Values were graphed using Prism software (GraphPad, Boston, MA) to fit a Scatchard plot where the saturation bound value (Bmax) was obtained for each case and then as average AD and control values. Plasma protein binding of 3H-**1** in lot-specific FBS was determined using 6 nM radioligand binding at ambient temperature followed by loading a 0.5 mL aliquot of FBS into an Amicon Ultra-0.5 mL 30 K purification device, which was centrifuged at 14,000 × g for 30 min at room temperature. The filtrate was then saved and the remaining supernatant was recovered by centrifugation at 1000×*g* for 2 min. The filtrate, recovered supernatant and filter itself were primed with ScintiVerse scintillation cocktail and were counted separately in a Beckman Coulter LS 6500 (Brea, CA) multi-purpose scintillation counter. The filtrate represented “free” or unbound radiotracer while the supernatant represented protein bound radiotracer (≥ 30 kDa protein-bound). The filter device represented non-specific binding and this value was subtracted from both supernatant and filtrate values. Percent free radiotracer was then used as a correction factor for measured Bmax values. A two-sided, two sample t-test was applied to Bmax averages between AD and control groups for each subregion as well as gray and white matter.

### Supplementary Information

Supplementary Material 1.

## Data Availability

The datasets used and/or analyzed during the current study are available from the corresponding author on reasonable request.

## Abbreviations

AD: Alzheimer’s dementia
CSF1R: Colony stimulating factor 1 receptor
LPS: Lipopolysaccharaide
PET: Positron emission tomography
SUV: Standardized uptake value
IPC: Inferior parietal cortex
CNS: Central nervous system
GBq: Giga becquerels
EOS: End-of-synthesis
SUVR: SUV_ratio_
FCTX: Frontal cortex
HIPP: Hippocampus
GM: Grey matter
WM: White matter
TSPO: Translocator protein
MCI: Mild cognitive impairment
GI: Gastrointestinal
TLC: Thin-layer chromatography
HPLC: High pressure liquid chromatography
ESI MS: Electrospray ionization mass spectrometry
PET/CT: PET combined with CT
PET/MRI: PET combined with MRI
FDx: Functional diagnosis
CERAD: Consortium to establish a registry for Alzheimer’s dementia
PMD: Post-mortem delay
P2X7: P2 purinergic receptor 7
MAO-B: Monoamineoxidase B
COX-1: Cyclooxygenase-1
COX-2: Cyclooxygenase-2
% ID/g: Percent injected dose per gram of tissue
MCI: Mild cognitive impairment
Iba1: Ionized calcium binding adapter molecule 1
pTau: PhosphoTau
GI: Gastrointestinal
