# Supplementary material for: A radioligand for in vitro autoradiography of CSF1R in post-mortem CNS tissues
Source: EJNMMI Res. 2024 Aug 26;14:76. doi: 10.1186/s13550-024-01133-2 (PMC11347546; doi:10.1186/s13550-024-01133-2)

**Supplemental Information**

Foss et al.

Mouse protocols:

Brain regional distribution of [^11^C]**1** in control mice, baseline: All animal studies were conducted within the guidelines of approved IACUC protocols for mice and non-human primates. Male CD1 mice of four to eight weeks of age, weighing 24-29 g from Charles River Laboratories (Wilmington, MA) were used. Animals were sacrificed by cervical dislocation at 3, 30 and 60 min (3 mice per time-point) following injection of 3.7 MBq (0.1 mCi) [^11^C]**1** [specific radioactivity = 348 GBq/μmol (9.4 Ci/μmol)] in 0.2 mL saline into a lateral tail vein. The brains were removed and dissected on ice. The brain regions (cerebellum, hippocampus, frontal cortex, brain stem and rest of brain) were weighed and their radioactivity content was determined in a γ-counter LKB/Wallac 1283 CompuGamma CS (Bridgeport, CT). The percentage of injected dose per gram of tissue %ID/g tiss was calculated (Table 2).

Brain regional distribution of [^11^C]**1** in neuroinflammation mouse model: The study was performed in the murine LPS-induced neuroinflammation model described previously[19]. Eighteen male CD1 mice ) were divided in three cohorts: 1) control mice (n = 6), baseline; 2) lipopolysaccharide (LPS) - IP treated (n = 6) mice, baseline; and 3) lipopolysaccharide (LPS) - IP treated (n = 6) mice, blocking with CPPC. The LPS (O111:B4, Calbiochem, San Diego, CA) solution in sterile saline (10 mg/kg, 0.2 mL) was administered intraperitoneally and the radiotracer study was performed on the 3rd day thereafter. The blocker, 0.1 mL CPPC solution in 20% DMSO and 80% saline (1 mg/kg), was given IP, 5 min before IV [11C]1, whereas baseline animals received vehicle.

The animals were injected IV with 3.7 MBq (0.1 mCi) [^11^C]**1** [specific radioactivity = 444 GBq/µmol (12 Ci/µmol)] and sacrificed by cervical dislocation at 45 min after the radiotracer injection. The whole brains were removed, dissected on ice and blood samples (0.2-0.5 cc) were taken from heartand their radioactivity content was determined in a γ-counter LKB/Wallac CompuGamma. The outcome variables were calculated as SUVR = SUVwhole brain/SUVblood.

**Supplemental Figures**

**
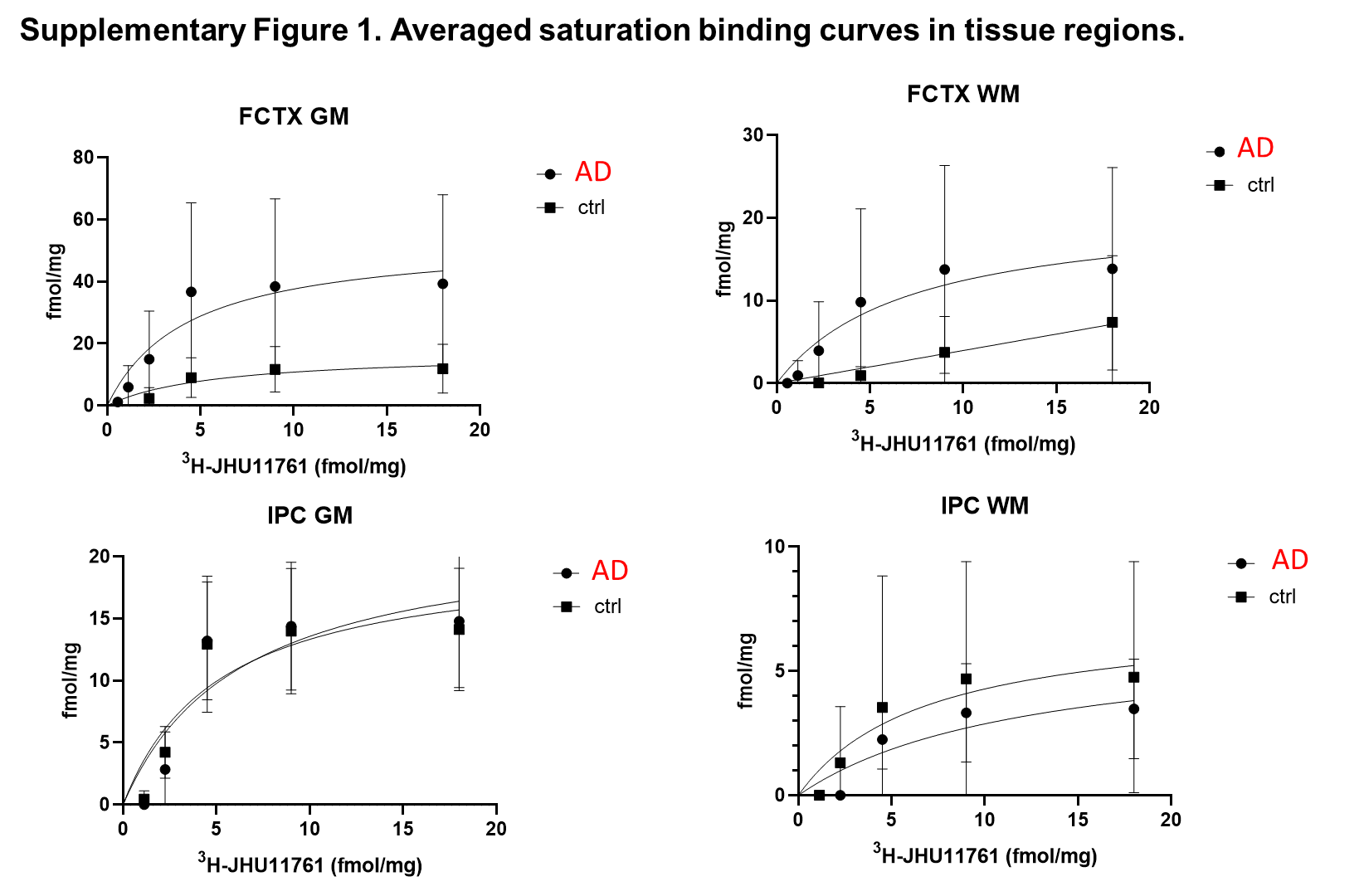
**

**
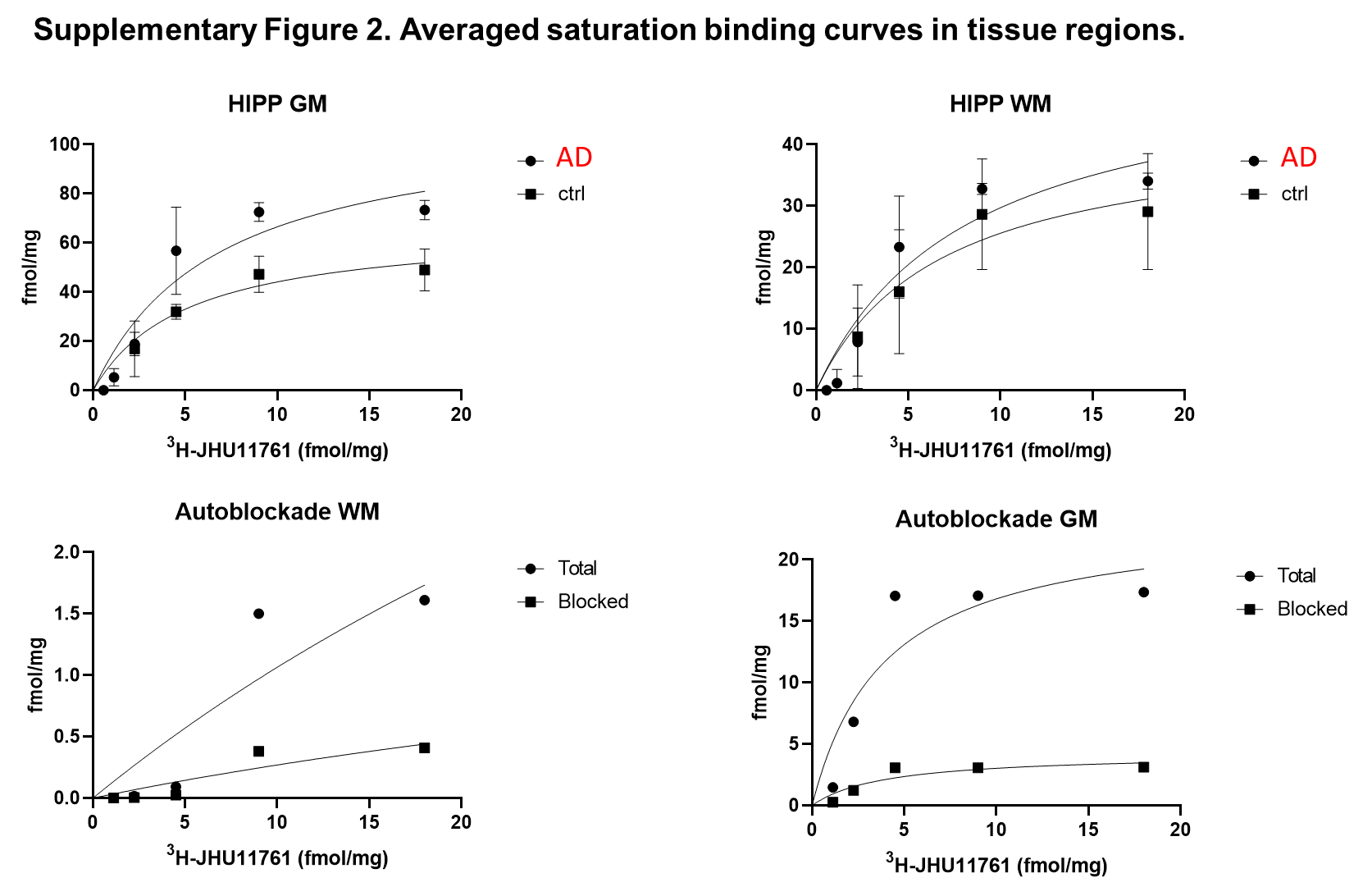
**


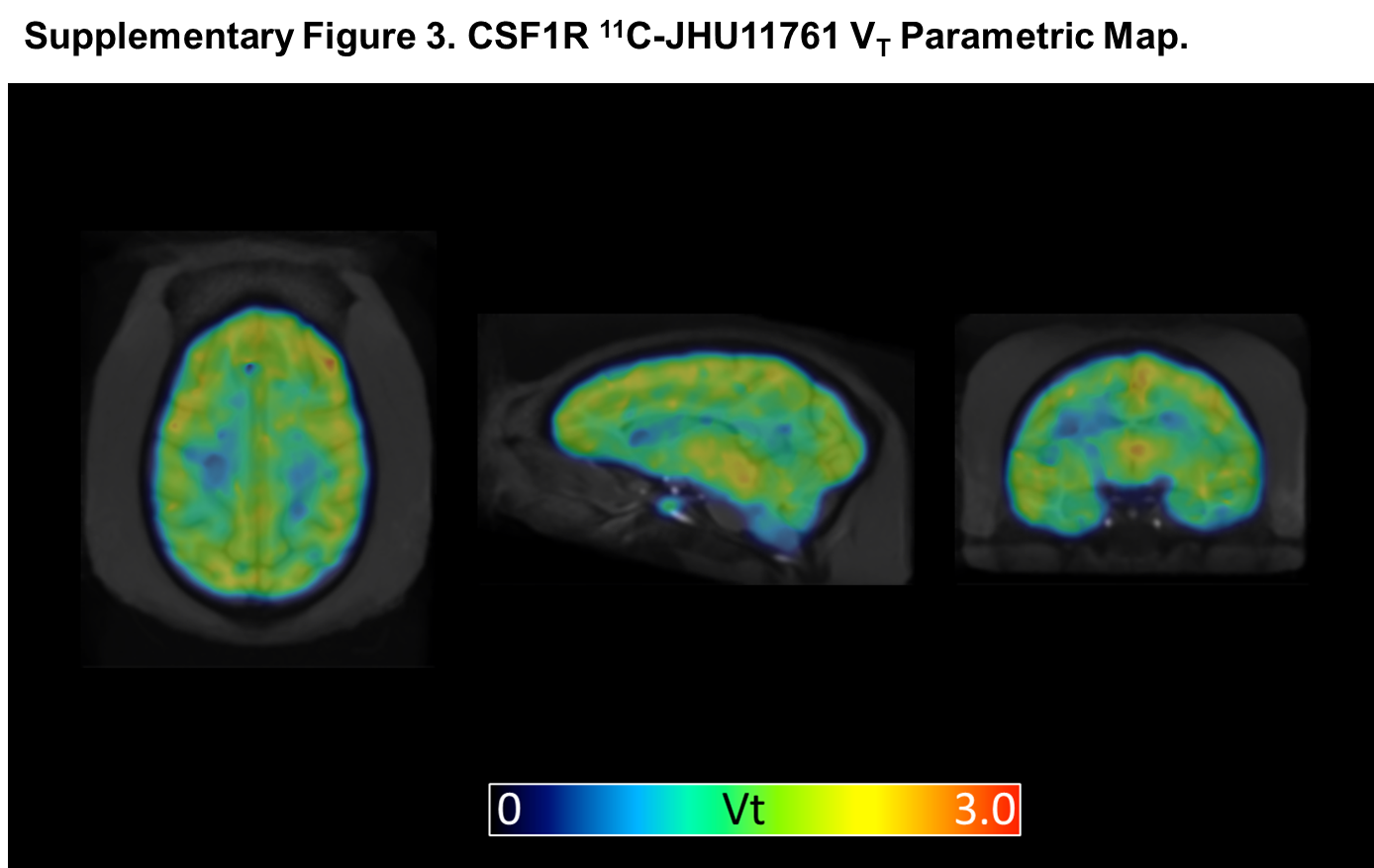

Supplement: Supplementary file 1 — Supplementary Material 1. [file 13550_2024_1133_MOESM1_ESM.docx]
